# Assembly, Core Microbiota, and Function of the Rhizosphere Soil and Bark Microbiota in *Eucommia ulmoides*

**DOI:** 10.3389/fmicb.2022.855317

**Published:** 2022-05-03

**Authors:** Chunbo Dong, Qiuyu Shao, Yulian Ren, Wei Ge, Ting Yao, Haiyan Hu, Jianzhong Huang, Zongqi Liang, Yanfeng Han

**Affiliations:** ^1^Institute of Fungus Resources, Department of Ecology, College of Life Sciences, Guizhou University, Guiyang, China; ^2^Analysis and Test Center, Huangshan University, Huangshan, China; ^3^State Key Laboratory of Environmental Geochemistry, Institute of Geochemistry, Chinese Academy of Sciences, Guiyang, China; ^4^Engineering Research Centre of Industrial Microbiology, Ministry of Education, Fujian Normal University, Fuzhou, China; ^5^Key Laboratory of Plant Resource Conservation and Germplasm Innovation in Mountainous Region (Ministry of Education), Guizhou University, Guiyang, China

**Keywords:** *Eucommia ulmoides* Oliv, plant-microbe interactions, community assembly, core microbiota, microbial functions

## Abstract

Medicinal plants are inhabited by diverse microbes in every compartment, and which play an essential role in host growth and development, nutrient absorption, synthesis of secondary metabolites, and resistance to biological and abiotic stress. However, the ecological processes that manage microbiota assembly and the phenotypic and metabolic characteristics of the core microbiota of *Eucommia ulmoides* remain poorly explored. Here, we systematically evaluated the effects of genotypes, compartment niches, and environmental conditions (climate, soil nutrition, and secondary metabolites) on the assembly of rhizosphere soil and bark associated bacterial communities. In addition, phenotypic and metabolic characteristics of *E. ulmoides* core microbiota, and their relationship with dominant taxa, rare taxa, and pharmacologically active compounds were deciphered. Results suggested that microbiota assembly along the two compartments were predominantly shaped by the environment (especially pH, relative humidity, and geniposide acid) and not by host genotype or compartment niche. There were 690 shared genera in the rhizosphere soil and bark, and the bark microbiota was mainly derived from rhizosphere soil. Core microbiota of *E. ulmoides* was a highly interactive “hub” microbes connecting dominant and rare taxa, and its phenotypic characteristics had a selective effect on compartment niches. Metabolic functions of the core microbiota included ammonia oxidation, nitrogen fixation, and polyhydroxybutyrate storage, which are closely related to plant growth or metabolism. Moreover, some core taxa were also significantly correlated with three active compounds. These findings provide an important scientific basis for sustainable agricultural management based on the precise regulation of the rhizosphere soil and bark microbiota of *E. ulmoides*.

## Introduction

Under natural conditions, plant growth and behavior are strongly dependent on related microbial communities called microbiome (Agler et al., 2016; Trivedi et al., 2020). These microorganisms inhabit various compartments of plant, including root, stems, leave, flower, and fruit, and influence host physiology and fitness by providing plants with nutrients and improving their resistance to biological and abiotic stresses (Mendes et al., 2013; Agler et al., 2016; Li et al., 2019). The phyllosphere and rhizosphere are vital interfaces for the interaction between host and environment and perform important biological functions in plants (Beckers et al., 2017). The rhizosphere microbiota is regarded as the second genome of plants, controlling the activation, uptake and utilization of nutrients in plants (Shen et al., 2021). Plant rhizosphere microbiota have functions similar to animal gut microbiota, which can not only regulate host performance local space, but also affect the host remotely and across spatial scales (Hou et al., 2021). Bark, an important organ of the plant, is always exposed to the oligotrophic environment with various temperatures and UV radiation (Dong et al., 2021c). It plays many roles in transporting nutrients and preventing the pests and diseases (Pellitier et al., 2019), however, the functions and construction processes of its associated microbiota are poorly understood.

*Eucommia ulmoides* Oliv., a medicinal and edible plant that produces an abundance of active compounds, is the source of *E. ulmoides* rubber used in industry and has high economic value. The bark contains pinoresinol diglucoside, geniposide acid, aucubin, chlorogenic acid, and other compounds with important clinical effects (He et al., 2014). Moreover, the gutta percha from *E. ulmoides* rubber is an excellent polymer material that contributes to the supply of rubber resources and the sustainable development of the rubber industry (Wei et al., 2021). Previous studies revealed that *E. ulmoides* bark harbored members of the microbial genera *Cladosporium*, *Sphingomonas*, *Alternaria*, *Devosia*, *Marmoricola*, *Rhodococcu*s, and *Teratosphaeria*, which were affected by climatic conditions, altitude, and secondary metabolites (Dong et al., 2020, 2021a,c). However, most of the related studies mainly considered one or a few aspects. We are still working to develop a systematic understanding of how compartment niches, host genotypes, soil factors, and microbial metabolites affect the construction of the *E. ulmoides* microbiota.

Studies suggested that plant-associated microbiota was predominantly determined by niche and host species/genotypes (Wagner et al., 2016; Cregger et al., 2018; Qian et al., 2019). Host genetic characteristics are an important driving force for the assembly of the microbiota. Composition and structure of the microbiota was affected by vertical transmission of endophytic or seed microbes, especially in the early stages of plant growth (Tkacz et al., 2015; Wagner et al., 2016; Xiong et al., 2021), but conversely, plants can also exert strong selective effects on their microbes through their own immune system, genetic network, and secondary metabolites (Cordovez et al., 2019). In addition, different compartments of plant were confirmed to have strong selectivity for the microbiota (Bai et al., 2015). For example, the phyllosphere community mainly comprises bacteria belonging to Proteobacteria, Bacteroidetes, and Firmicutes, where members of Proteobacteria constitute ∼50% of the community composition (Trivedi et al., 2020). In addition to this biological pressure, assembly of the plant-associated microbiota is also controlled by climate, soil properties, and human disturbance (Carlström et al., 2019).

Not all members of the microbiota are beneficial to plants, and a significant proportion of microbiota members are neutral and even a small number are harmful (Berg, 2009; Mendes et al., 2013). Particular microbes, termed “hub microbes or core microbes” due to their central position in a microbial network, are disproportionally important in shaping microbial communities on plant hosts (Agler et al., 2016). The core microbiota of medicinal plants may be instrumental in determining the quality of medicinal materials (Huang et al., 2018; Dong et al., 2021a; Xu et al., 2021). The core microbiota not only directly play a beneficial role (Chen et al., 2018) but can also influence the wider microbial community through the community cascade effect, thereby driving evolution and function of the microbial community (Agler et al., 2016). The bark of *E. ulmoides* was reported to be rich in fungal diversity, especially the rare fungal taxa that cannot be ignored (Dong et al., 2021c). Increasing numbers of studies have suggested that the relationship between rare taxa and ecosystem functions may be closer than previously expected (Pascoal et al., 2021; Zhao et al., 2022). Nevertheless, the function of the core microbiota and its relationship with dominant microbiota and rare microbiota has yet to be elucidated.

This study selected 66 *E. ulmoides* samples (33 rhizosphere soil and 33 bark samples) from 11 regions with obvious geographical distribution. We aimed to assess how host (compartment niches and genotypes) and environmental conditions (longitude and latitude, altitude, climate, soil nutrition, and metabolites) interactively shape assembly of microbiota with different compartments of *E. ulmoides*; and to explore the composition and function of the core microbiota of *E. ulmoides*.

## Materials and Methods

### Soil Rhizosphere and Bark Sampling

A total of 66 soil rhizosphere and bark samples from 11 representative regions (Zunyi, Panzhou, Wangcang, Heishui, Cili, Liuyang, Shuanglong, Shiyan, Linbao, Qimen, and Longshi) of *E. ulmoides* in China were used in the study (Supplementary Figure 1). Three healthy *E. ulmoides*, approximately 10 m from one another, were selected in each region, and bark samples from 1.5 m above the ground of each tree were collected (Dong et al., 2020). Sub-samples for chemical analysis were transported to the laboratory, dried, and crushed, followed by the measurement of pinoresinol diglucoside, aucubin, and geniposidic acid by tandem liquid chromatography quadrupole time of flight mass spectrometry (LC-QTOF MS/MS). Sub-samples for DNA extraction were frozen in dry ice during transportation and stored at −80°C until further processing. Subsequent chemical analysis and DNA extraction were performed as described previously Dong et al. (2020).

Similarly, three healthy *E. ulmoides* trees were chosen from each region, and rhizosphere soil sample was collected from each tree. For each tree, rhizosphere soil samples from three vertical directions (approximately 1 m away from the trunk) at a depth of 20 cm were collected, and were uniformly mixed into a sample (Xu et al., 2018). Soil samples were used to extract the total genomic DNA and measure physiochemical factors.

### DNA Extraction, PCR Amplification, and Illumina MiSeq Sequencing of Rhizosphere Soil

Total genomic DNA of *E. ulmoides* rhizosphere soil samples was extracted according to FastDNA Spin Kit for Soil (MP, United States). In this study, DNA concentration between 40 and 60 ng/μL was considered qualified. According to universal primer pair 338F (5′-ACTCCTACGGGAGGCAGCAG-3′) and 806R (5′-GGACTACHVGGGTWTCTAAT-3′), the 16S rRNA gene was amplified. The conditions and mixtures for PCR amplification referred to our previous method (Dong et al., 2021b). PCR products were sequenced by Majorbio Bio-Pharm Technology Co. Ltd. (Shanghai, China) based on an Illumina MiSeq PE300 platform (Illumina, San Diego, United States). Most of these analysis were performed on BMK Cloud.^1^ The raw data was uploaded to the NCBI database (BioProject ID: PRJNA771480; BioSample: SAMN22311478).

### Amplicon Data of Bark Samples

The bark sampling method was according to our previous study (Dong et al., 2021b), and the current work is a follow-up study to explore the construction mechanism of the rhizosphere soil and bark microbiota and the function of the core microbiota. The bark sample was corresponding to the rhizosphere soil sample. The diversity of bark bacterial communities was characterized based on universal primer pair 338F and 806R. The sequencing data of *E. ulmoides* bark (BioProject ID: PRJNA771480; BioSample: SAMN22311479) was collated and compiled based on our previous study (Dong et al., 2021b).

### Data of Host Genotype, Active Compounds, and Soil Physicochemical Properties

The approach of genotype characteristics, active compounds, and soil physicochemical properties were described in our previous study (Dong et al., 2021b). The genetic characteristics of *E. ulmoides* were analyzed based on the chloroplast gene trnH-psbA (forward: 5′-CGCGCATGGTGGATTCACAATCC-3′; Reverse: 5′-GTTATGCATGAACGTAATGCTC-3′) and trnL-F (forward: 5′-ATTTGAACTGGTGACACGAG-3′; Reverse: 5′-CGAAATCGGTAGACGCTACG-3′), and these analyses were performed by the software on BioEdit v7.0.9.0, MAFFT v 7.037, MEGA6, SequenceMatrix1.7.8, and DnaSP version 6. The content of active compounds was determined based on LC-QTOF MS/MS. The determination of soil physicochemical properties mainly refers to “Physical and Chemical Analysis of Soil Properties.” In this study, the data of host genotypes, active compounds, and soil physicochemical properties were placed in Supplementary Tables 1, 2.

### Definition of the Core Microbiota, Dominant Microbiota, and Rare Microbiota

Core microbiota was defined with reference to the method of Dong et al. (2021a). The operational taxonomic units (OTUs) with all samples were screened out to construct a co-occurrence network. Highly significant (r > 0.6 or r < −0.6; *p* < 0.05) spearman correlations between bacterial co-occurrence network were visualized by Gephi, version 0.9.2. Similarly, network topology parameters (e.g., degree, betweenness centrality, closeness centrality, hub, clustering, and modularity) were calculated in the software Gephi, version 0.9.2. In the study, the threshold for core microbiota identification was degree ≥ 20, and closeness centrality ≥ 0.29. In addition, OTUs with relative abundance above 0.1% were placed within the dominant taxa (Xiong et al., 2021), while rare taxa refer to OTUs with relative abundance below 0.01% (Pascoal et al., 2021).

### Phenotypic and Functional Analysis of Core Microbiota

The habitat, energy source, and metabolic functions of the core microbiota were annotated based on METAGENassist^2^ (Arndt et al., 2012). The phenotypic information of 11,000 microorganisms and the full-sequence of approximately 1,800 microorganisms were included in METAGENassist. Moreover, the phenotypic information contains approximately 20 categories for each microorganism, such as metabolism, habitat, energy source(s), oxygen requirements, preferred temperature range.

### Data Analysis

The weighted UniFrac distance matrix was calculated to evaluate beta-diversity (β-diversity), and then non-metric multidimensional scaling (NMDS) was used for ranking analysis (Xiong et al., 2021). The significance of different factors on community dissimilarity was tested with permutational multivariate analysis of variance (PERMANOVA) or nested PERMANOVA using the “adonis” function of the VEGAN package in R 3.6.3 (Oksanen et al., 2015). Null model analysis was performed “picante” package (Zhao et al., 2021), Structural equation model was analyzed the “lavaan” package in R 3.6.3 (Rosseel, 2012). Traceability analysis refers to the fast expectation-maximization for microbial source tracking (FEAST), which is a ready-to-use scalable framework that can simultaneously estimate the contribution of thousands of potential source environments in a timely manner, thereby helping unravel the origins of complex microbial communities^3^ (Shenhav et al., 2019).

## Results

### Environmental Conditions Determine Assembly of the Rhizosphere Soil and Bark Microbiota of *Eucommia ulmoides*

**FIGURE 1:**
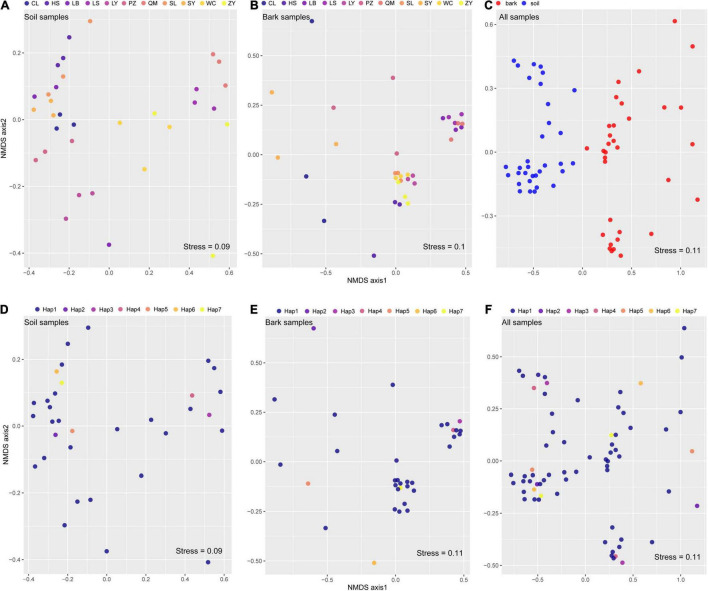
Microbiome assembly was shaped more strongly by environmental conditions than by host niches or genotypes of *E. ulmoides*. Nonmetric multidimensional scaling (NMDS) ordinations based on weighted UniFrac distances matrices of bacterial communities for: **(A)** rhizosphere soil (*n* = 33); **(B)** bark (*n* = 33); **(C)** all samples of different niches (*n* = 66); **(D)** soil samples of different genotypes (*n* = 33); **(E)** bark samples of different genotypes (*n* = 33); **(F)** all samples of different genotypes (*n* = 33).

Non-metric multidimensional scaling and PERMANOVA showed that variation of bacterial communities in the rhizosphere soil (*R*^2^ = 78.84%, *p* = 0.001) and bark (*R*^2^ = 67.14%, *p* = 0.001) of *E. ulmoides* was predominantly determined by environmental conditions (Figures 1A,B and Table 1). The compartment niche did not have a significant effect on *E. ulmoides*-associated bacterial communities (*R*^2^ = 1.73%, *p* = 0.28) (Table 1), but the rhizosphere soil and bark samples clustered clearly and independently (Figure 1C and Supplementary Figure 2). Similarly, different genotypes had no significant effects on the rhizosphere soil and bark bacteria of *E. ulmoides* (Figures 1D–F and Table 1).

**TABLE 1:** The influences of host niche, genotype, and environmental factors on the bacterial community of *E. ulmoides* based on PERMANOVA.

Variable	All samples				Soil samples				Bark samples			
	*F* value	df	*R*^2^ (%)	Pr (>*F*)	*F* value	df	*R*^2^ (%)	Pr (>*F*)	*F* value	df	*R*^2^ (%)	Pr (>*F*)
Niches	1.13	1	1.73	0.28	na	na	na	na	na	na	na	na
Environments	na	na	na	0.77	8.2	10	78.84	0.001	4.5	10	67.14	0.001
Genotypes	0.68	6	6.49	0.961	0.73	6	14.4	0.931	1.17	6	21.29	0.156

### Active Compounds, Soil Physicochemical Properties, and Climate Factors Affect the Microbial Diversity of Rhizosphere Soil and Bark

**FIGURE 2:**
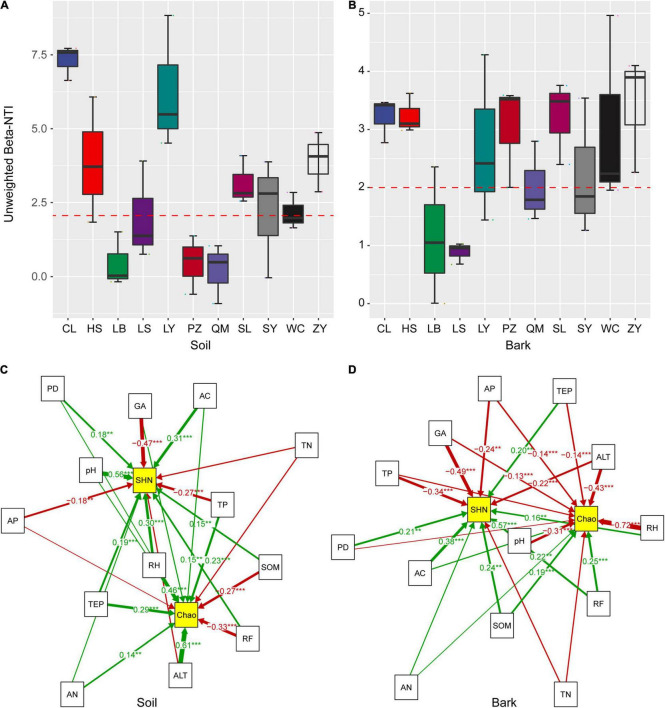
Null model and structural equation model analysis reveals the bacterial community of assembly process of *E. ulmoides*. β-NTI values of bark **(A)** and rhizosphere soil **(B)** bacterial communities were distributed in 11 producing areas **(C)**, Fitting relationships between active compounds, soil physicochemical factors, and climatic factors, and diversity index of soil bacterial community. **(D)** Fitting relationships between active compounds, soil physicochemical factors, and climatic factors, and diversity index of bark bacterial community. Active compounds included geniposidic acid (GA), aucubin (AC), and pinoresinol diglucosid (PD). Soil physicochemical factors included available phosphorus (AP), soil organic matter (SOM), pH, total phosphorus (TP), available nitrogen (AN), and total nitrogen (TN). Climatic factors included Rainfall (RF), relative humidity (RH), altitude (ALT), and temperature (TEP). Diversity index includes Shannon diversity index (SHN), Chao diversity index (Chao). Solid lines and arrows show significant and non-significant relationships between different variables (variables with normalized path coefficients > 0.1). Significance levels were **p < 0.01, ***p < 0.001.

According to the unweighted β-NTI statistics, the β-NTI values of the rhizosphere soil and bark bacterial communities of *E. ulmoides* mainly ranged from −1 to 9 (Figures 2A,B), suggesting that the assembly of bacterial community was affected by both stochastic and deterministic factors (β-NTI value > 2, then the deterministic process was dominant; β-NTI value < 2, then the stochastic process was dominant). SEM further suggested that the bacterial α-diversity in the rhizosphere soil and bark of *E. ulmoides* was affected to varying degrees by host active components, soil physicochemical properties, and climatic factors (Figures 2C,D). For example, pH (with an estimated value of 0.56, *p* < 0.001), aucubin (0.31, *p* < 0.001), and relative humidity (0.30, *p* < 0.001) had significant positive effects on the Shannon diversity index, while geniposide acid had a significant negative effect on the Shannon diversity index (−0.47, *p* < 0.001). Altitude (0.61, *p* < 0.001) and relative humidity (0.46, *p* < 0.001) had significant positive effects on the Chao index, while rainfall had a significant negative effect on the Chao index (Figure 2C).

The bark and rhizosphere soil microbiota respond similarly to environmental factors. pH (0.57, *p* < 0.001) and aucubin (0.38, *p* < 0.001) had significant positive effects on the Shannon diversity index; geniposide acid (−0.49, *p* < 0.001) and total phosphorus (−0.34, *p* < 0.001) had significant negative effects on the Shannon diversity index; and pH (−0.72, *p* < 0.001), altitude (−0.43, *p* < 0.001), and relative humidity (−0.31, *p* < 0.001) had significant negative effects on the Chao index (Figure 2D). Overall, the composition and structure of the *E. ulmoides* microbiota were driven by multiple environmental factors.

### Differences and Potential Source Tracking of Rhizosphere Soil and Bark Bacterial Communities

**FIGURE 3:**
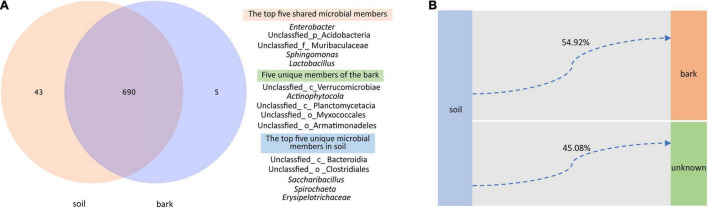
Genus-level differences and potential source tracking of rhizosphere soil and bark bacterial communities. **(A)** Venn diagrams showing shared and unique bacterial genera in different niches. **(B)** FEAST showing that the rhizosphere soil bacterial community was a potential source of the bark bacterial community.

Although compartment niches were not the main factor affecting the microbiota of *E. ulmoides*, there were still similarities and differences in the composition of the microbial communities of rhizosphere soil and bark. At the genus level, 690 taxa were shared between rhizosphere soil and bark microbial communities, among which the top five in relative abundance were Enterobacter, an unidentified genus of Acidobacteria, an unidentified genus of Muribaculaceae, *Sphingomonas*, and Lactobacillus. Bark contained five unique taxa, which were all unidentified genera of Verrucomicrobiae, Actinophytocola, Planctomycetacia, Myxococcales, and Armatimonadeles. In contrast, rhizosphere soil contained 43 unique taxa, among which the top five relative abundances comprised unidentified genera of Bacteroidia, Clostridiales, and *Saccharibacillus*, *Spirochaeta*, and Erysipelotrichaceae (Figure 3A).

Fast expectation-maximization microbial source tracking (FEAST) suggested that 54.92% of the members of the bark bacterial community were derived from rhizosphere soil, and 45.08% of the members were derived from other unknown environments (Figure 3B).

### Composition of Core Microbiota and Its Relationship With Dominant Microbiota and Rare Microbiota

**FIGURE 4:**
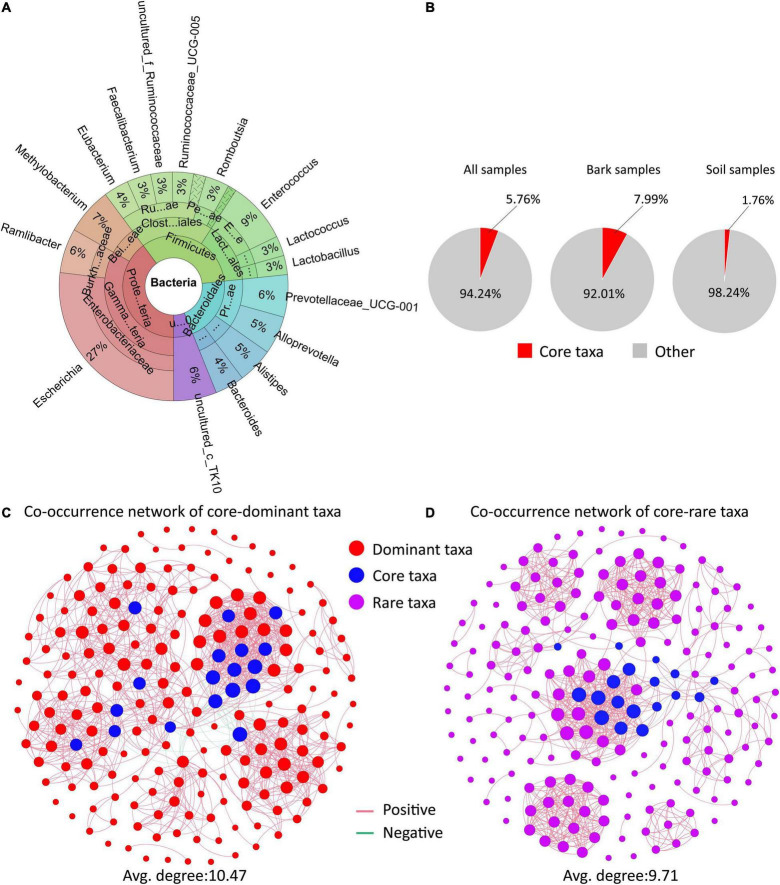
The interactions of the core microbiota with the rare microbiota. **(A)** Composition and classification information of the core microbiota. **(B)** Distribution of core microbiota in different samples. **(C,D)** Bacterial co–occurrence networks along the core taxa–dominant microbiota **(C)** and core taxa–rare microbiota **(D)**.

Here, we identified dominant taxa, core taxa and rare taxa (Figures 4A,B and Supplementary Figures 3, 4). A total of 201 OTUs (0.1% of total OTU number) were defined as the dominant microbiota, with *Enterobacter*, *Actinobacteria*, and *Sphingomonas* containing the most OTUs. The dominant microbiota accounted for 78.89% of the soil samples, and 70.61% of the bark samples (Supplementary Figure 4a). A total of 185 OTUs (0.01% of total OTU number) were defined as rare microbiota, of which the Proteobacteria Deltaproteobacteria, Burkholderiaceae, and Rhizobiales contained the most OTUs (Supplementary Figure 4b). Based on the network parameters, 18 OTUs were defined as core microbiota (Supplementary Figure 3), and these belonged to the genera *Escherichia*, *Enterococcus*, *Methylobacterium*, and *Ramlibacter*. The proportion of core microbiota was higher in bark samples and lower in soil samples compared to the dominant microbiota (Figures 4A,B).

To further characterize the impact of the core microbiota on other members of the host microbiota, potential interrelationships between the core microbiota and the dominant and rare microbiota were evaluated (Figures 4C,D). There was no significant difference in the complexity of the two microbial networks (average degree representing network complexity). Within co-occurrence networks, we found that correlations were usually positive (core-dominant taxa, 97.35%, *n* = 1057; core-rare, 99.1%, *n* = 783) (Figures 4C,D). Core microbiota was a highly interactive “hub” microbes connecting dominant and rare taxa, play an important role in determining microbiome structures.

### Phenotypic and Functional Characteristics of Core Microbiota

**FIGURE 5:**
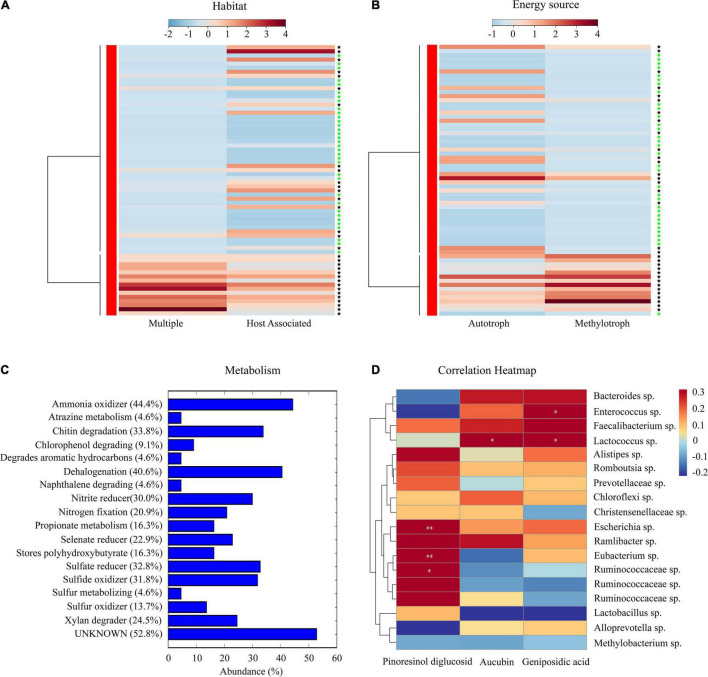
Phenotypic characteristics and potential metabolic functions of the core microbiota of *E. ulmoides*. **(A)** Habitat information of the core microbiota. **(B)** Energy source of the core microbiota. **(C)** Potential metabolism of the core microbiota. **(D)** Heatmap of the correlation between core microbiota and active compounds. The “black asterisk” represents rhizosphere soil samples, “green asterisk” represents bark samples. Significance levels were *p < 0.05, ^**^p < 0.01.

Phenotypic and functional characteristics of the core microbiota of *E. ulmoides* were deciphered based on the METAGENassist platform and statistical analysis (Figure 5). The core microbiota with multiple habitat characteristics mostly exists in the soil rhizosphere, while the core microbiota related to the host predominantly exist in the bark of *E. ulmoides*. Based on energy source utilization, the core microbiota present in the rhizosphere soil were mainly methylotrophs, while the core microbiota existing in the bark were mostly autotrophs (Figure 5).

Metabolic functions of the core microbiota of *E. ulmoides* predominantly comprised ammonia oxidizer, dehalogenation, chitin degradation, sulfate reducer, and sulfide oxidizer. In addition, some metabolic functions closely related to the biological properties of *E. ulmoides* were worthy of attention, such as xylan degrader, nitrogen fixation, and stores polyhydroxybutyrate (Figure 5C). The correlation heatmap showed that *Escherichia*, *Eubacterium*, and an unidentified genus of the family Ruminococcaceae were significantly correlated with pinoresinol diglucoside, *Lactococcus* exhibited a significant correlation with aucubin, and *Enterococcus* and *Lactococcus* were significantly correlated with geniposidic acid (Figure 5).

## Discussion

The microbiota of medicinal plants is an important factor affecting the health and productivity of the host (Maggini et al., 2020), and can be directly or indirectly involved in the production of biologically active compounds by the host (Koberl et al., 2013). The medicinal plant-associated microbiota has been gradually characterized with the omics technology (Chen et al., 2018; Ulloa-Muñoz et al., 2020; Dong et al., 2021c; Sauer et al., 2021). However, the mechanism of medicinal plant-associated microbiota construction and the phenotype and function of the core microbiota have yet to be elucidated. Findings from the current study demonstrated that assembly of the rhizosphere soil and bark microbiota was shaped predominantly by environmental factors, rather than by host genotype or compartment niche. Moreover, the core microbiota were key taxonomic units for the construction of microbial networks and were closely related to the growth and the accumulation of secondary metabolites of *E. ulmoides*.

### Multiple Environmental Factors Influence Diversity of the Microbial Community of *Eucommia ulmoides*

Assembly of plant-associated microbiota is regulated by the host’s metabolism, genotype, soil physicochemical properties, climate, and niche (Wagner et al., 2016; Cregger et al., 2018; Sun et al., 2020; Xiong et al., 2021). In the current study, assembly of the rhizosphere soil and bark microbiota was predominantly shaped by environmental factors and not by genotype or compartment niche (Table 1). Environmental factors are generally accepted to be closely related to the composition of plant-associated microbiota (Breidenbach et al., 2015; Trivedi et al., 2020; Zhang et al., 2020; Bona et al., 2021). Relative humidity was significantly correlated with the diversity index of the soil and bark bacterial communities, which was consistent with previous studies (Dong et al., 2021c). As reported by Cruz-Paredes et al. (2021), environmental humidity within a certain range significantly impacts the respiration and growth of microorganisms. Moreover, pH could also significantly affect assembly of microbial communities in soil and bark (Figure 2). This may be because pH combines multiple soil chemical variables and is the result of many chemical reactions between organic (biological and abiotic) and inorganic soil components (Lopes et al., 2021). The bark microbiota was not directly in contact with soil pH but was still significantly affected by pH in the current study (Figure 2B). It was previously reported that the indirect impact of soil pH on the microbiota in other compartments of the plant is because microbes in the other compartments were derived from rhizosphere soil (Fitzpatrick et al., 2018; Lopes et al., 2021). Similarly, FEAST analysis suggested that 54.92% of the members of the bark bacterial community were derived from rhizosphere soil (Figure 3B).

Venn diagram analysis showed that 690 genera, such as *Enterobacter*, *Sphingomonas*, and *Lactobacillus* (Figure 3A), were shared between soil and bark communities. These genera were also found in previous studies on *E. ulmoid*es (Dong et al., 2020, 2021a) and are beneficial microorganisms with potential ecological functions for medicinal plants (Quijada et al., 2018; Asaf et al., 2020; Webster et al., 2020). Highly abundant unique bacteria of the rhizosphere soil samples included Bacteroidia, Clostridiales, *Saccharibacillus*, *Spirochaeta*, and *Erysipelotrichaceae*, which have specific selective effects on the soil, predominantly exist in the soil, and play an important role in geo-biological-chemical circulation (Wilms et al., 2006; Namirimu et al., 2019). Most of the bacteria unique to the bark are those with relatively older evolutionary status, such as the Planctomycetes, which are important for the nitrogen cycle (Cavalier-Smith, 2002). Myxococcus, which is only detected in the bark of rhododendrons, has broad application potential in agriculture, biomedicine, and environmental protection due to its ability to produce a variety of natural products (Wang et al., 2021).

### The Core Microbiota Is a Strain Reservoir Rich in Medicinal Secondary Metabolites of *Eucommia ulmoides*

Core microbiota, an important subset of the host-associated microbiota, can not only promote the growth of host plants but also recruit functional species and block pathogens/pests (Banerjee et al., 2018; Hassani et al., 2018; Toju et al., 2018). In recent years, the core microbiota of medicinal plants has been accepted as a processing plant or reservoir for secondary metabolites (Koberl et al., 2013; Chen et al., 2018; Xu et al., 2021). Using microbial network analysis, 18 core bacteria of *E. ulmoides* belonging to the genera *Escherichia*, *Enterococcus*, *Methylobacterium*, *Eubacterium*, and *Lactococcus* were identified (Figure 4A). Among them, species of *Escherichia*, *Eubacterium*, and Ruminococcaceae were significantly related to pinoresinol diglucoside; a species of Lactococcus had a significant correlation with aucubin; and species of *Enterococcus* and *Lactococcus* were significantly correlated with geniposide acid (Figure 4D). Previous studies have shown that *Escherichia* sp. (Leonard et al., 2008), *Enterococcus* sp. (Müller et al., 2001), *Eubacterium* sp. (Newman and Cragg, 2020), and *Lactococcus* sp. (Golomb and Marco, 2015), Ruminococcaceae sp. (Yin et al., 2020) could promote plant growth or accumulate secondary metabolites. The phenotypic information indicated that the core microbiota mainly contained methylotrophic bacteria in the rhizosphere soil and mostly autotrophic bacteria in the bark (Figure 5B). This indicated that the core microbiota from the rhizosphere soil may play the role of consumers, while the core microbiota from the bark may predominantly play producer roles (Thakur and Geisen, 2019; El-Sayed et al., 2021). The metabolome of the 18 core bacteria was further analyzed, and in addition to some common metabolic functions of the plant microbiota (Xu et al., 2018; Trivedi et al., 2019), the functions of xylan degradation, nitrogen fixation, and polyhydroxybutyrate storage were worthy of additional attention. Xylan-degrading and nitrogen-fixing bacteria were reported to potentially exhibit direct or indirect effects on plant growth and metabolism (Gopalakrishnan et al., 2017; Jung et al., 2018). Polyhydroxybutyrate was a type of polyhydroxyalkanoate (PHA), a polyester polymer (Sirohi et al., 2021) that has similar structure and function to *E. ulmoides* rubber, such as strong plasticity (He et al., 2014; Sirohi et al., 2021). Therefore, the core taxa with polyhydroxybutyrate metabolism functions may be involved in the synthesis of *E. ulmoides* rubber. In general, these findings reveal that the core microbiota may participate in soil organic matter decomposition and metabolite synthesis in *E. ulmoides*.

### The Core Microbiota Is the Hub of the Assembly of the Associated-Microbiota of *Eucommia ulmoides*

Network analysis can be used to identify the core or hub microbiota at the center of the microbial network (Banerjee et al., 2018; Dong et al., 2021a). Core microbiota is composed by important taxonomic units that dominate host microbial community construction, influencing community structure through strong interactions with the host or other microbes rather than simply being present in high abundance (Banerjee et al., 2018; Hamonts et al., 2018; Trivedi et al., 2020; Dong et al., 2021c). A comprehensive investigation of the *Arabidopsis* phyllosphere microbiota found that the core microorganisms *Albugo* sp. and *Dioszegia* sp. regulate the entire microbial community by inhibiting the growth and diversity of other microbes (Agler et al., 2016). The current study found that the core microbiota was highly connected with the dominant microbiota, forming closely related microbial clusters (Figure 4C). Dominant microbiota and core microbiota have vital ecological roles in microbiota assembly and ecosystem functions (Banerjee et al., 2018). Core microbiota can selectively adjust and assist these dominant microbiota (Banerjee et al., 2018), such as by forming a highly specific microbiota with the dominant microbiota for biological nitrogen fixation (Fierer, 2017). In contrast with the dominant microbiota, rare microbiota was often overlooked. Recent studies confirmed that rare microbiota was instrumental in the construction, function, and stability of microbiota (Jousset et al., 2017; Dong et al., 2021c; Pascoal et al., 2021). Similarly, significant interactions between core and rare microbiota of *E. ulmoides* were detected in the current study (Figure 4D). This is congruent with previous findings that rare microbiota often interacts with other species although they occupy narrow ecological niches (Fierer et al., 2007). Together these data suggest the core microbiota not only has important functions but may also be a keystone species that determines the network structure of the *E. ulmoides* microbial community.

## Data Availability Statement

The datasets presented in this study can be found in online repositories. The names of the repository/repositories and accession number(s) can be found in the article/Supplementary Material.

## Author Contributions

YH, TY, and JH: conceptualization and funding acquisition. CD, QS, YR, and WG: data acquisition. CD and HH: formal analysis. QS: writing the first draft. CD, YH, and ZL: writing, review, and editing the manuscript. All authors have read and agreed to the published version of the manuscript.

## Conflict of Interest

The authors declare that the research was conducted in the absence of any commercial or financial relationships that could be construed as a potential conflict of interest.

## Publisher’s Note

All claims expressed in this article are solely those of the authors and do not necessarily represent those of their affiliated organizations, or those of the publisher, the editors and the reviewers. Any product that may be evaluated in this article, or claim that may be made by its manufacturer, is not guaranteed or endorsed by the publisher.

## Footnotes

1
https://console.biocloud.net/

2
http://www.metagenassist.ca

3
https://github.com/cozygene/FEAST

## Supplementary Material

Supplementary Figure 1 Geographic distribution of 11 regions of *E. ulmoides* in China.

Supplementary Figure 2 Hierarchical clustering of rhizosphere soil and bark samples of *E. ulmoides* based on the unweighted paired average method (UPGMA).

Supplementary Figure 3 The core microbiota of *E. ulmoides* was defined based on the network connection method. **(a)** The topology of the correlation co-occurrence network. **(b)** Core microbiota were defined based on closeness centrality and degree.

Supplementary Figure 4 The composition and distribution of dominant **(a)** and rare taxa **(b)**.
