# Supplementary material for: Assembly, Core Microbiota, and Function of the Rhizosphere Soil and Bark Microbiota in Eucommia ulmoides
Source: Front Microbiol. 2022 May 3;13:855317. doi: 10.3389/fmicb.2022.855317 (PMC9110929; doi:10.3389/fmicb.2022.855317)
Supplement: Supplementary file 1 [file Table_1.DOCX]

**Table S1** Haplotypes, microbial diversity and active compounds information of *E. ulmoides* from different regions.

| Sample | Group | Haplotype | Chao | Shannon | Pinoresinol diglucosid (μg/mL) | Aucubin (μg/mL) | Geniposidic acid(μg/mL) |
| --- | --- | --- | --- | --- | --- | --- | --- |
| ZY1 | Zunyi | Hap1 | 1249.068 | 7.0279 | 8.35 | 18.88 | 23.38 |
| ZY2 | Zunyi | Hap1 | 11445.16 | 6.9153 | 22.76 | 31.63 | 31.02 |
| ZY3 | Zunyi | Hap1 | 1288.074 | 7.7076 | 24.51 | 14.23 | 17.39 |
| PZ1 | Panzhou | Hap1 | 901.5769 | 7.9294 | 24.6 | 16.67 | 18.25 |
| PZ2 | Panzhou | Hap1 | 1060.12 | 7.7997 | 16.34 | 32.17 | 21.62 |
| PZ3 | Panzhou | Hap1 | 1457.558 | 8.7871 | 13 | 11.84 | 1.55 |
| WC1 | Wangcang | Hap1 | 1349.143 | 7.806 | 24.83 | 21.56 | 30.8 |
| WC2 | Wangcang | Hap1 | 1324.167 | 7.9585 | 22.43 | 17.83 | 28.81 |
| WC3 | Wangcang | Hap1 | 1144.433 | 7.7236 | 31.39 | 31.11 | 20.77 |
| HS1 | Heishui | Hap9 | 1038.177 | 7.4274 | 27.51 | 1.33 | 11.12 |
| HS2 | Heishui | Hap1 | 1380 | 7.0797 | 14.16 | 7.52 | 13.06 |
| HS3 | Heishui | Hap1 | 1184.933 | 7.1746 | 22.58 | 0.97 | 8.46 |
| CL1 | Cili | Hap3 | 1111.82 | 7.8863 | 32.85 | 36.53 | 39.54 |
| CL2 | Cili | Hap8 | 1101.455 | 7.9612 | 27.12 | 28.48 | 35.57 |
| CL3 | Cili | Hap1 | 942.1707 | 7.9948 | 24.92 | 5.88 | 24.49 |
| LY1 | Liuyang | Hap1 | 1236.897 | 7.9491 | 11.83 | 7.22 | 6.94 |
| LY2 | Liuyang | Hap1 | 1475.9 | 8.2451 | 18.58 | 7.37 | 9.38 |
| LY3 | Liuyang | Hap1 | 1174.714 | 7.1924 | 13.86 | 6.5 | 15.46 |
| SL1 | Shuanglong | Hap1 | 1152.222 | 7.7324 | 19.49 | 39.29 | 26.5 |
| SL2 | Shuanglong | Hap1 | 1207.138 | 7.6081 | 23.55 | 36.29 | 25.14 |
| SL3 | Shuanglong | Hap1 | 1380.667 | 7.9574 | 33.2 | 41.77 | 36.45 |
| SY1 | Shiyan | Hap12 | 967.9355 | 6.0505 | 10.25 | 1.14 | 0.52 |
| SY2 | Shiyan | Hap1 | 964.5882 | 7.7491 | 19.87 | 41.5 | 8.47 |
| SY3 | Shiyan | Hap1 | 1165.41 | 7.5522 | 23.28 | 7.02 | 5.64 |
| LB3 | Linbao | Hap1 | 1287 | 8.0869 | 33.29 | 4.6 | 12.53 |
| LB4 | Linbao | Hap1 | 1219.037 | 8.2969 | 30.51 | 8.47 | 9.24 |
| LB5 | Linbao | Hap1 | 1431.143 | 8.3354 | 20.54 | 6.45 | 7.09 |
| QM1 | Qimen | Hap1 | 1144.955 | 8.3632 | 26.32 | 47.43 | 30.95 |
| QM2 | Qimen | Hap1 | 1409.75 | 8.5039 | 30.48 | 46.65 | 25.35 |
| QM3 | Qimen | Hap1 | 1188.64 | 8.3117 | 35.11 | 16.69 | 20.66 |
| LS1 | Longshi | Hap4 | 1297.909 | 8.3943 | 19.49 | 39.29 | 26.5 |
| LS2 | Longshi | Hap5 | 1210.182 | 8.2567 | 23.55 | 36.27 | 25.14 |
| LS3 | Longshi | Hap1 | 1212.5 | 8.4532 | 33.2 | 41.77 | 36.45 |

**Table S2** Soil physicochemical and climatic information of *E. ulmoides* from different regions.

| Sample | Group | pH | AN(mg/kg) | SOM(mg/kg) | AP(mg/kg) | TN(g/kg) | TP(g/kg) | Relative humidity (%) | Temperature (℃) | Rainfall (mm) | Altitude (m) |
| --- | --- | --- | --- | --- | --- | --- | --- | --- | --- | --- | --- |
| ZY1 | Zunyi | 5.47 | 97.28 | 6.21 | 8.41 | 1.79 | 0.2315 | 85.911 | 15.696 | 1049.253 | 1002.25 |
| ZY2 | Zunyi | 5.35 | 95.26 | 6.16 | 11.73 | 1.9 | 0.2088 | 85.911 | 15.696 | 1049.253 | 1002.25 |
| ZY3 | Zunyi | 5.25 | 94.27 | 6.23 | 13.29 | 2.01 | 0.2224 | 85.911 | 15.696 | 1049.253 | 1002.25 |
| PZ1 | Panzhou | 7.12 | 209.88 | 11.01 | 27.5 | 4.83 | 0.2527 | 74.842 | 15.271 | 1170.163 | 1525.47 |
| PZ2 | Panzhou | 7.03 | 211.68 | 10.9 | 23.91 | 4.95 | 0.2497 | 74.842 | 15.271 | 1170.163 | 1525.47 |
| PZ3 | Panzhou | 6.98 | 207.93 | 10.87 | 24.87 | 5.07 | 0.2219 | 74.842 | 15.271 | 1170.163 | 1525.47 |
| WC1 | Wangcang | 6.22 | 110.69 | 0.89 | 9.92 | 1.91 | 0.2002 | 82.454 | 15.732 | 1239.095 | 427.35 |
| WC2 | Wangcang | 6.35 | 119.95 | 0.95 | 10 | 2.02 | 0.2048 | 82.454 | 15.732 | 1239.095 | 427.35 |
| WC3 | Wangcang | 6.13 | 116.25 | 0.98 | 11.3 | 2.13 | 0.2128 | 82.454 | 15.732 | 1239.095 | 427.35 |
| HS1 | Heishui | 6.68 | 199.57 | 19.92 | 31.69 | 2.51 | 0.4186 | 76.448 | 8.34 | 799.926 | 2203.63 |
| HS2 | Heishui | 6.45 | 201.8 | 20.86 | 33.26 | 2.47 | 0.3838 | 76.448 | 8.34 | 799.926 | 2203.63 |
| HS3 | Heishui | 6.81 | 204.36 | 22.01 | 32.68 | 2.43 | 0.3974 | 76.448 | 8.34 | 799.926 | 2203.63 |
| CL1 | Cili | 6.01 | 150.14 | 0.98 | 6.38 | 3.02 | 0.12 | 86.901 | 17.725 | 1382.543 | 74.56 |
| CL2 | Cili | 6.02 | 141.12 | 0.95 | 6.51 | 2.94 | 0.118 | 86.901 | 17.725 | 1382.543 | 74.56 |
| CL3 | Cili | 6.14 | 146.35 | 1.1 | 10.37 | 2.86 | 0.1216 | 86.901 | 17.725 | 1382.543 | 74.56 |
| LY1 | Liuyang | 5.42 | 111.63 | 7.44 | 13.15 | 2.31 | 0.5412 | 91.283 | 18.047 | 1545.66 | 67.12 |
| LY2 | Liuyang | 5.34 | 109.37 | 7.59 | 18.38 | 2.2 | 0.5251 | 91.283 | 18.047 | 1545.66 | 67.12 |
| LY3 | Liuyang | 5.38 | 109.58 | 7.68 | 15.54 | 2.09 | 0.5402 | 91.283 | 18.047 | 1545.66 | 67.12 |
| SL1 | Shuanglong | 7.73 | 70.39 | 6.01 | 20.66 | 1.95 | 0.4368 | 74.216 | 10.079 | 652.929 | 977.96 |
| SL2 | Shuanglong | 7.68 | 70.56 | 5.22 | 36.25 | 1.85 | 0.4111 | 74.216 | 10.079 | 652.929 | 977.96 |
| SL3 | Shuanglong | 7.71 | 73.1 | 5.33 | 17.9 | 1.75 | 0.4792 | 74.216 | 10.079 | 652.929 | 977.96 |
| SY1 | Shiyan | 6.95 | 65.21 | 8.29 | 66.17 | 2.93 | 0.9053 | 78.108 | 16.163 | 782.922 | 202.04 |
| SY2 | Shiyan | 6.91 | 63.5 | 8.06 | 73.83 | 3.02 | 0.9104 | 78.108 | 16.163 | 782.922 | 202.04 |
| SY3 | Shiyan | 6.89 | 62.94 | 8.11 | 62.95 | 3.11 | 0.9099 | 78.108 | 16.163 | 782.922 | 202.04 |
| LB3 | Linbao | 7.67 | 186.54 | 6.24 | 28.21 | 2.07 | 0.4489 | 73.43 | 11.898 | 722.167 | 977.22 |
| LB4 | Linbao | 7.68 | 184.16 | 6.16 | 29.29 | 2.12 | 0.4519 | 73.43 | 11.898 | 722.167 | 977.22 |
| LB5 | Linbao | 7.59 | 189.29 | 6.17 | 26.51 | 2.17 | 0.4423 | 73.43 | 11.898 | 722.167 | 977.22 |
| QM1 | Qimen | 5.47 | 334.39 | 9.91 | 21.76 | 3.03 | 0.6118 | 96.329 | 14.141 | 2239.47 | 84.04 |
| QM2 | Qimen | 5.45 | 338.69 | 9.48 | 20.7 | 2.94 | 0.6032 | 96.329 | 14.141 | 2239.47 | 84.04 |
| QM3 | Qimen | 5.57 | 339.87 | 9.24 | 20.93 | 2.85 | 0.6032 | 96.329 | 14.141 | 2239.47 | 84.04 |
| LS1 | Longshi | 5.41 | 194.36 | 8.99 | 27.12 | 2.33 | 0.1801 | 92.002 | 16.918 | 1654.516 | 317.29 |
| LS2 | Longshi | 5.34 | 195.45 | 9.01 | 22.34 | 2.47 | 0.1871 | 92.002 | 16.918 | 1654.516 | 317.29 |
| LS3 | Longshi | 5.42 | 197.22 | 8.88 | 26.53 | 2.61 | 0.1821 | 92.002 | 16.918 | 1654.516 | 317.29 |
