# Supplementary figures and images for: Assembly, Core Microbiota, and Function of the Rhizosphere Soil and Bark Microbiota in Eucommia ulmoides
Source: Front Microbiol. 2022 May 3;13:855317. doi: 10.3389/fmicb.2022.855317 (PMC9110929; doi:10.3389/fmicb.2022.855317)

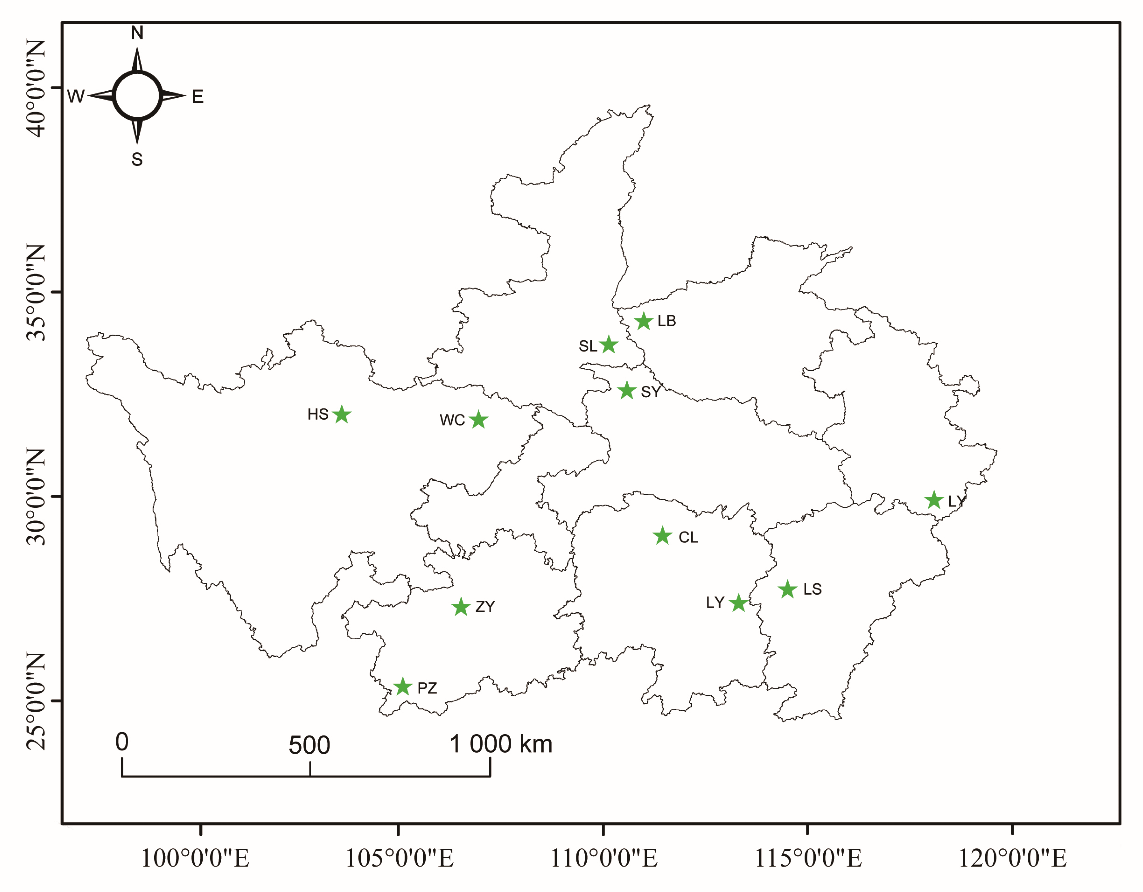

Supplement: Supplementary Figure 1 — Geographic distribution of 11 regions of E. ulmoides in China. [file Image_1.TIF]

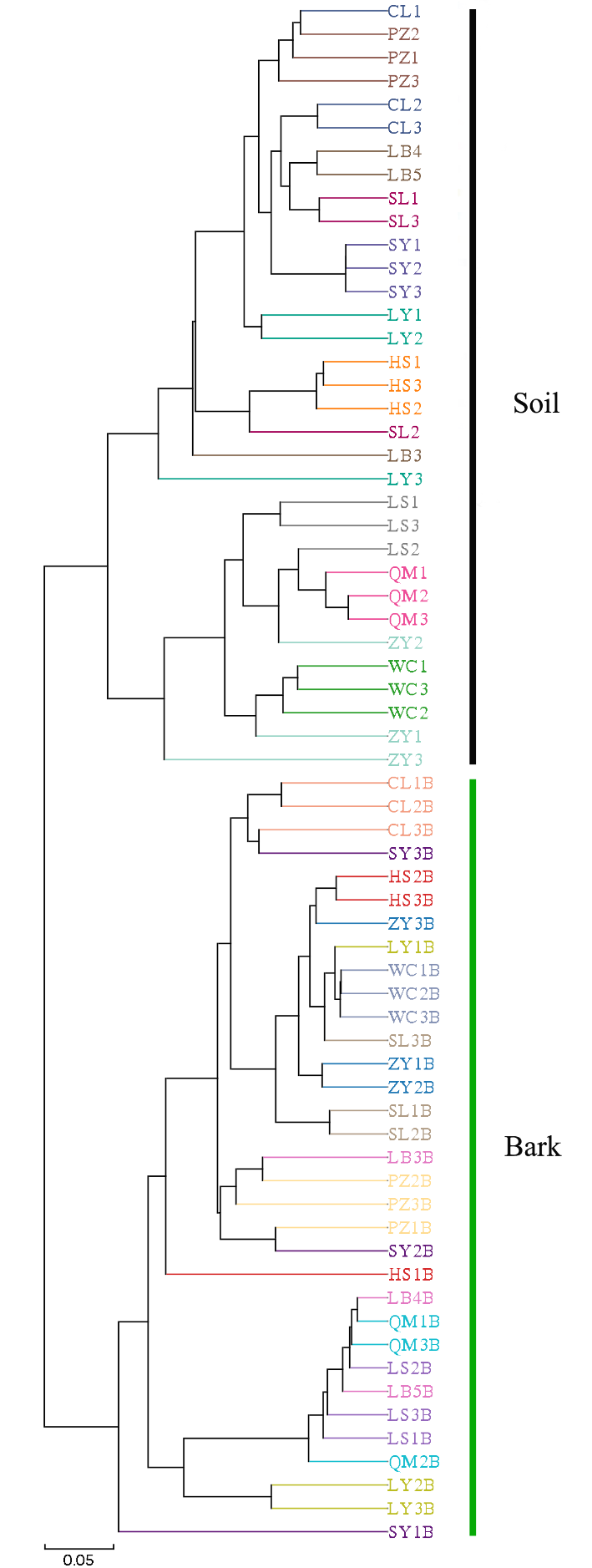

Supplement: Supplementary Figure 2 — Hierarchical clustering of rhizosphere soil and bark samples of E. ulmoides based on the unweighted paired average method (UPGMA). [file Image_2.TIF]

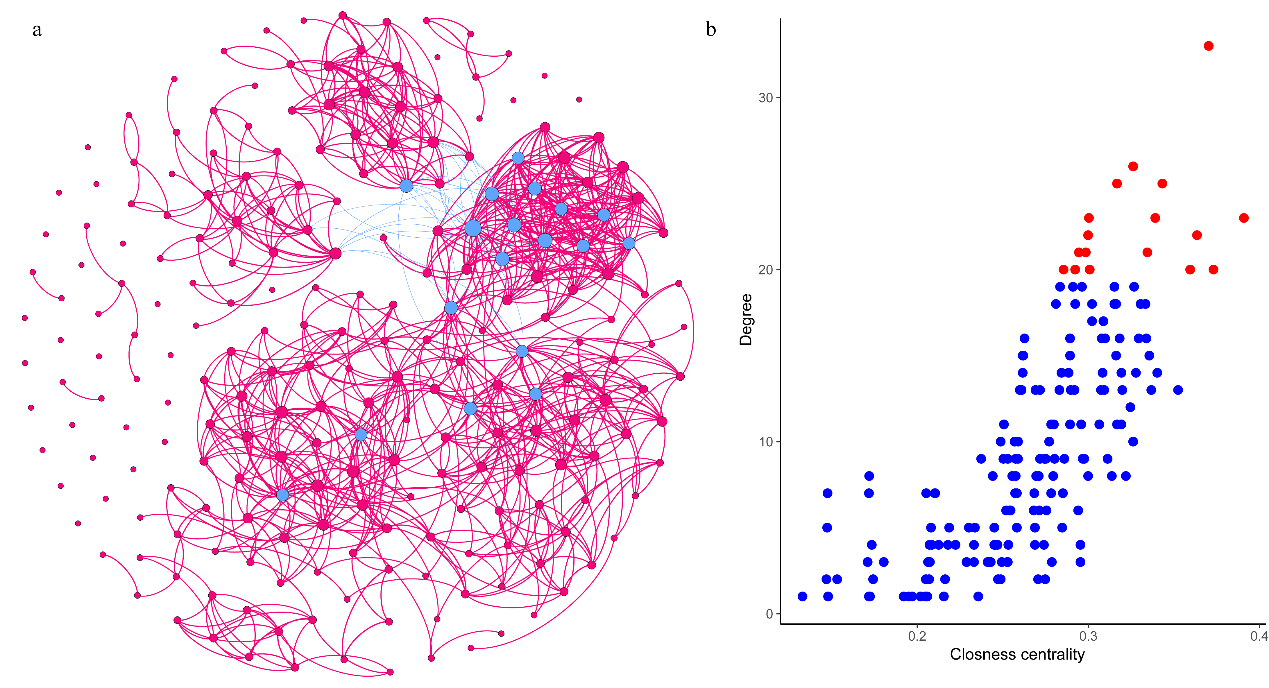

Supplement: Supplementary Figure 3 — The core microbiota of E. ulmoides was defined based on the network connection method. (a) The topology of the correlation co-occurrence network. (b) Core microbiota were defined based on closeness centrality and degree. [file Image_3.TIF]
